# Supplementary material for: dsRNA-Seq: Identification of Viral Infection by Purifying and Sequencing dsRNA
Source: Viruses. 2019 Oct 14;11(10):943. doi: 10.3390/v11100943 (PMC6832592; doi:10.3390/v11100943)
Supplement: Supplementary file 1 [file viruses-11-00943-s001.zip › viruses-578448-supplementary to xml/RevisedSupplemental/Figure_S1.pdf]

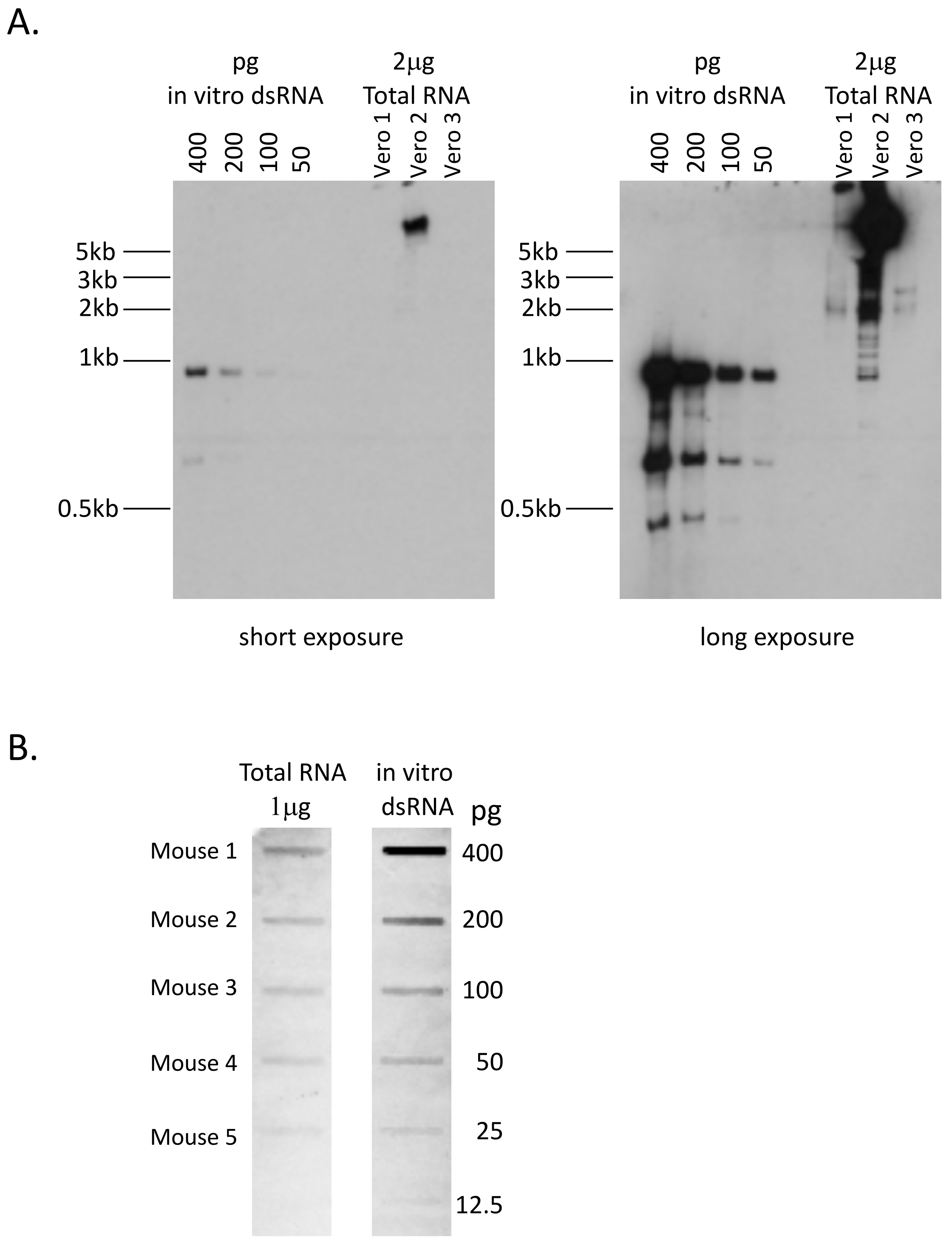

Figure S1. Immunoblot analysis of total RNA samples using anti-dsRNA antibody
Antibody against dsRNA was used to estimate the amount of dsRNA in total RNA isolated from Vero cell samples or mouse tissue samples. Immunoblotting was performed as described in [22, 25] (a) Blot of native acrylamide gel containing 2μg of total RNA from Vero cell samples or dilution series of known amount of in vitro transcribed 0.9kb dsRNA [22]. (b) 1μg of total RNA from each mouse total RNA sample was loaded in a slot blot and compared to a dilution series of in vitro transcribed 0.9kb dsRNA.
