# Supplementary material for: dsRNA-Seq: Identification of Viral Infection by Purifying and Sequencing dsRNA
Source: Viruses. 2019 Oct 14;11(10):943. doi: 10.3390/v11100943 (PMC6832592; doi:10.3390/v11100943)
Supplement: Supplementary file 1 [file viruses-11-00943-s001.zip › viruses-578448-supplementary to xml/RevisedSupplemental/Figure_S2.pdf]

A.

					% Recovery		
Sample #	Total RNA (µg)	dsRNA (ng)	single-strand RNase treatment	antibody	top strand dsRNA	bottom strand dsRNA	GAPDH mRNA
1	10	100	yes	anti-dsRNA	58	49	ND
2	10	10	yes	anti-dsRNA	102	52	ND
3	10	1	yes	anti-dsRNA	51	40	ND
4	10	0.1	yes	anti-dsRNA	170	151	ND
5	10	0.01	yes	anti-dsRNA	92	86	ND
6	10	1	no	anti-GFP	ND	ND	ND

ND=Not detected by qRT-PCR

B.

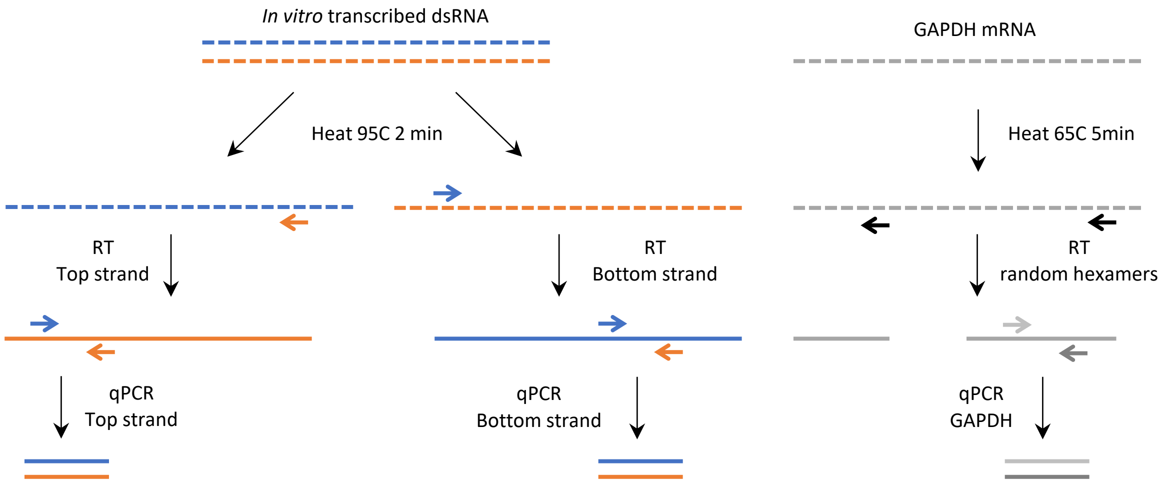

C.

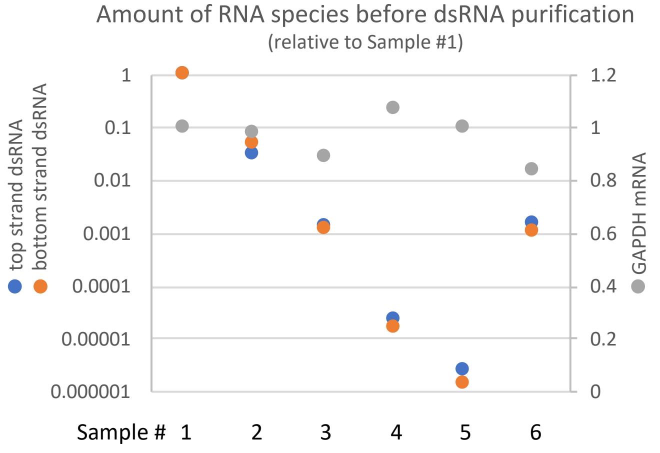

D.

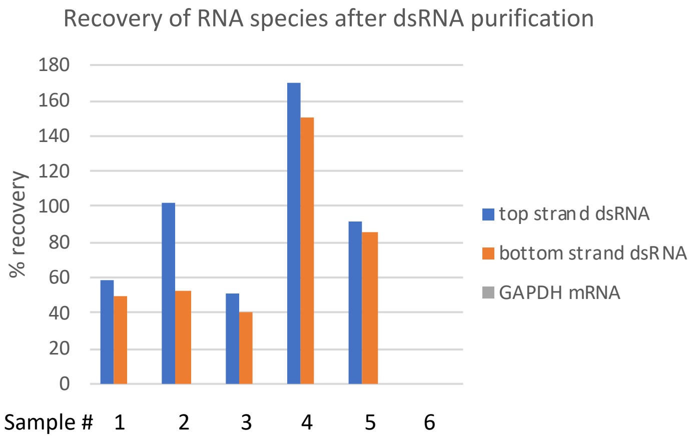

Figure S2 Analysis of dsRNA purification method.
(a) The two-step dsRNA purification method described in Materials and Methods was used to purify dsRNA from samples containing a fixed amount of total RNA isolated from human tissue culture cells spiked with varying amounts of a 0.9kb *in vitro* transcribed dsRNA [22]. The RNA samples were treated with or without single-strand specific RNase and then dsRNA was isolated using either 5μg of the anti-dsRNA antibody J2 or a control antibody against GFP.
(b) Schematic of the three qRT-PCR reactions used to determine the amount of the individual strands of the dsRNA or a single-strand RNA (GAPDH mRNA) in the samples before and after dsRNA purification. RNA indicated by dashed lines, DNA by solid lines. (c) Amount of the three RNA species in starting samples before dsRNA purification procedure plotted relative to Sample 1. Relative amount of dsRNA strands reported on left axis on log scale. Relative amount of GAPDH mRNA reported on right axis on linear scale. (d) Recovery of the three RNA species after dsRNA purification. In all samples, the recovery of GAPDH mRNA was below the detection limits of the qRT-PCR.
