# Supplementary material for: dsRNA-Seq: Identification of Viral Infection by Purifying and Sequencing dsRNA
Source: Viruses. 2019 Oct 14;11(10):943. doi: 10.3390/v11100943 (PMC6832592; doi:10.3390/v11100943)
Supplement: Supplementary file 1 [file viruses-11-00943-s001.zip › viruses-578448-supplementary to xml/RevisedSupplemental/Figure_S3.pdf]

A.

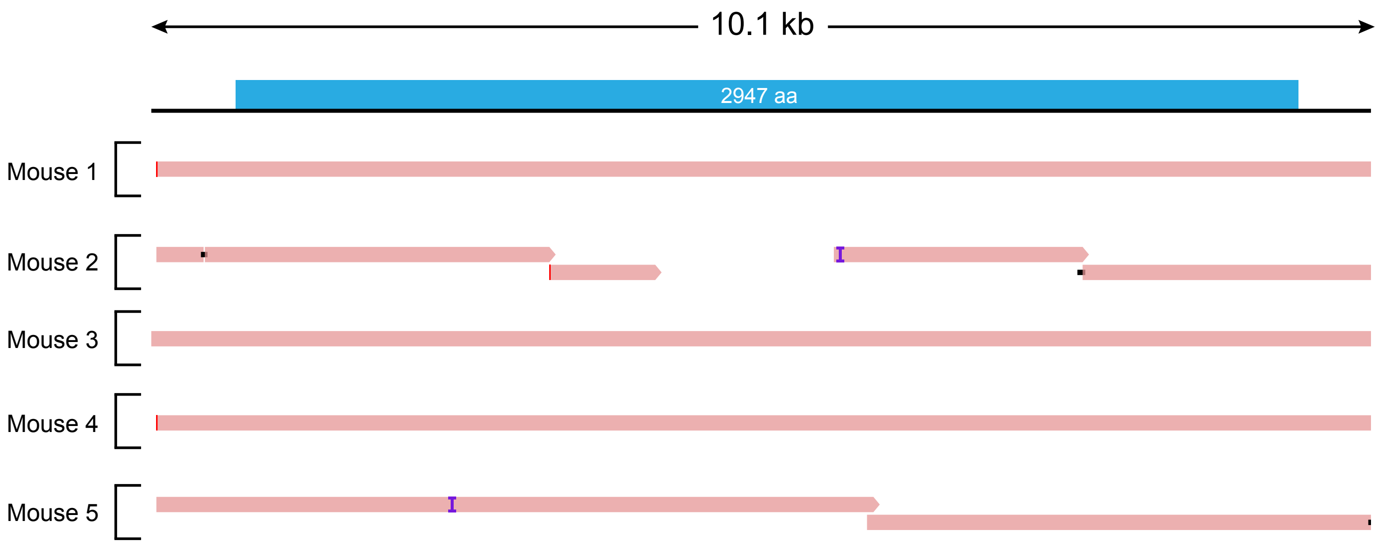
10.1 kb
2947 aa
Mouse 1
Mouse 2
Mouse 3
Mouse 4
Mouse 5

B.

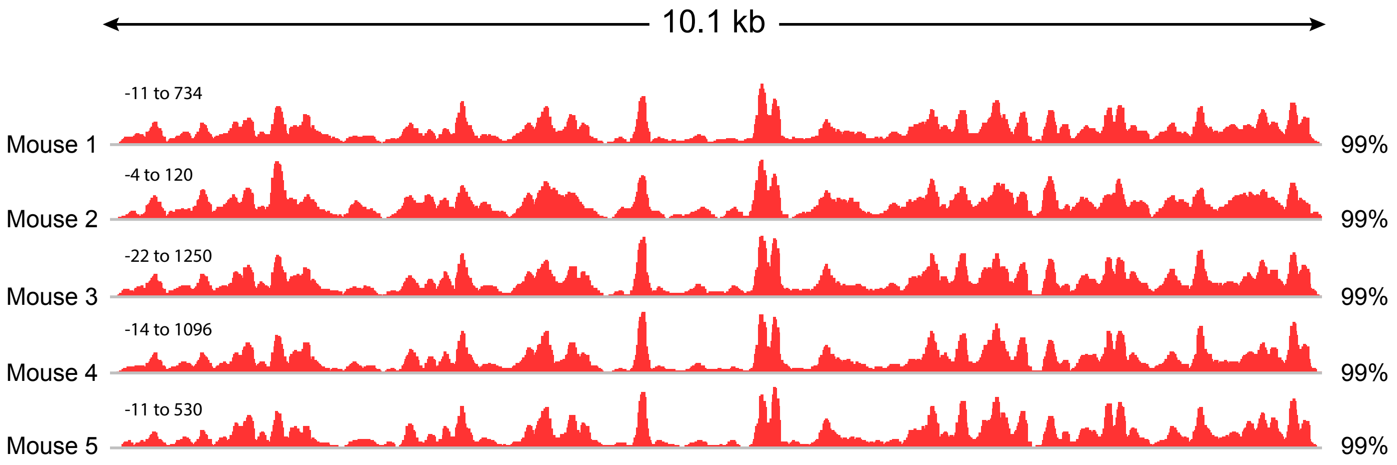
10.1 kb
-11 to 734
Mouse 1
99%
-4 to 120
Mouse 2
99%
-22 to 1250
Mouse 3
99%
-14 to 1096
Mouse 4
99%
-11 to 530
Mouse 5
99%

Figure S3

Figure S3. Picorna-like viral contigs assembled from mouse dsRNA-Seq libraries are derived from single-stranded RNA. (a) Alignment of contigs representing novel picorna-like virus. The longest contig with similarity to Sanxia picorna-like virus 7 (MM3_TRINITY_DN634_c0_g1_i5) was selected as a representative. Contigs assembled from each mouse dsRNA-Seq library were aligned to the representative contig using bwa-mem. The location of the long open reading frame present in the representative contig is illustrated at the top. (b) Bedgraphs indicating the base count of reads from dsRNA-Seq libraries that aligned to each position of the representative picorna-like virus contig. Red indicates counts on positive strand. Blue indicates counts on negative strand. The percentage of total reads that mapped to the representative picorna-like virus contig that aligned to the positive strand are indicated on the right. The bedgraphs were not scaled relative to total number of dsRNA-Seq reads in each sample. Each read set was scaled individually, scale is indicated at top left for each set.
