# Supplementary material for: dsRNA-Seq: Identification of Viral Infection by Purifying and Sequencing dsRNA
Source: Viruses. 2019 Oct 14;11(10):943. doi: 10.3390/v11100943 (PMC6832592; doi:10.3390/v11100943)
Supplement: Supplementary file 1 [file viruses-11-00943-s001.zip › viruses-578448-supplementary to xml/RevisedSupplemental/Figure_S4.pdf]

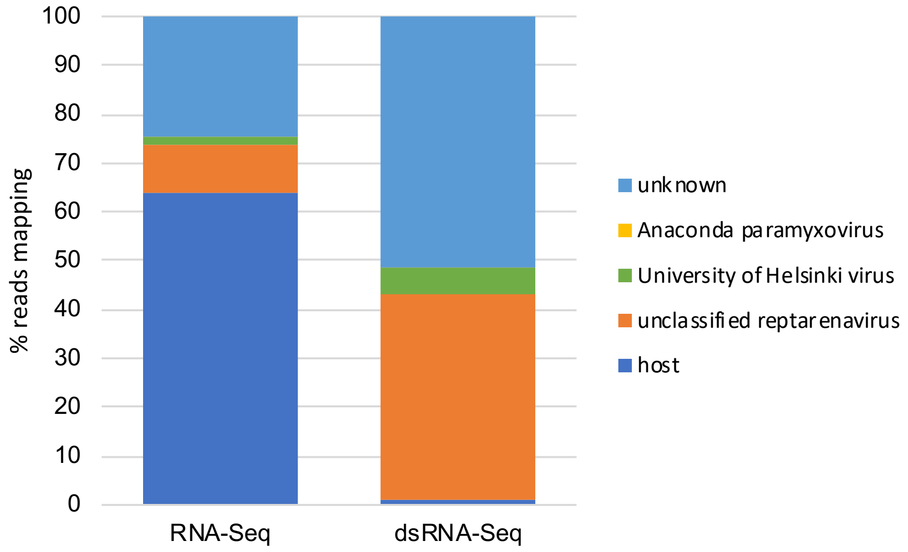

Figure S4 Mapping of dsRNA-Seq and RNA-Seq reads from boa constrictor kidney sample to host and viral sequences.

The percentage of total RNA-Seq and dsRNA-Seq reads from the boa constrictor kidney sample that aligned to boa constrictor genome scaffolds (host), dsRNA contigs with similarity to the unclassified reptarenavirus, dsRNA contigs with similarity to University of Helsinki virus and dsRNA contigs with similarity to Anaconda paramyxovirus. Reads that did not align to the genome scaffolds or the viral dsRNA contigs were assigned as unknown.
